# Preventative interventions by dental care professionals in Africa on oral human papillomavirus, gonorrheal, chlamydial, syphilitic and trichomonas infections: A scoping review

**DOI:** 10.1002/hsr2.1246

**Published:** 2023-05-02

**Authors:** Kehinde K. Kanmodi, Jimoh Amzat, Afeez A. Salami, Eyinade A. Egbedina, Ramat O. Braimah

**Affiliations:** ^1^ Faculty of Dentistry University of Puthisastra Phnom Penh Cambodia; ^2^ Campaign for Head and Neck Cancer Education (CHANCE) Programme Cephas Health Research Initiative Inc Ibadan Nigeria; ^3^ School of Health and Life Sciences Teesside University Middlesbrough UK; ^4^ Department of Sociology Usmanu Danfodiyo University Sokoto Nigeria; ^5^ Department of Sociology University of Johannesburg Johannesburg South Africa; ^6^ Department of Oral and Maxillofacial Surgery University College Hospital Ibadan Nigeria; ^7^ Department of Dental and Maxillofacial Surgery Usmanu Danfodiyo University Teaching Hospital Sokoto Nigeria

**Keywords:** Africa, dental care, interventions, scoping review, sexually transmitted diseases

## Abstract

**Background:**

Oral human papillomavirus (HPV), gonorrheal, chlamydial, syphilitic and trichomonas infections are very common sexually transmitted diseases (STDs) in Africa. However, no known study has reviewed the available evidence concerning the preventative interventions by dental care professionals (DCPs) in Africa on oral STDs; hence, this scoping review was conducted to evaluate the research landscape of this topic area in Africa.

**Methods:**

The scoping review methodology and documentation were informed by the Arksey and O'Malley's guideline, the Preferred Reporting Items for Systematic Reviews and Meta‐analysis extension for conducting Scoping Reviews (PRISMA‐ScR) checklist, and the AMSTAR‐2 guideline. Ten electronic research databases were searched to retrieve literatures relevant to the scoping review question. The retrieved literature were deduplicated and screened for eligibility based on the review's selection criteria. Data charting, collation and summarization were intended to be done in this review, but it could not be done because no relevant literature was found eligible for inclusion into this scoping review.

**Results:**

A total of 523 literature were retrieved. After deduplication of the retrieved literatures, the residual literatures (*n* = 353) were screened for eligibility for inclusion into the review, of which no eligible article was found. Hence, this scoping review was an empty review.

**Conclusion:**

This empty scoping review demonstrates that DCPs in Africa do not engage in research‐based oral STD prevention. Therefore, the implementation of research‐based preventative interventions, by DCPs, on oral STDs should be encouraged in Africa.

## 1 INTRODUCTION

Sexually transmitted diseases (STDs), previously referred to as sexually transmitted infections (STIs), are conditions caused by the transmission of a broad array of pathogens between sexual partners through diverse routes of sexual contact, such as anal, vaginal, or oral route.^1^ Sexually transmitted diseases has remained as an issue of global public health concern due to its close association with natural physiologic response which is “sex” and majority of persons affected with these diseases goes untreated with serious consequences.^2^ While these diseases are most prevalent in adolescents and young adults (due to high sexual activities), the most serious consequences occur later in life.^1^

The eight most common STDs include four incurable but treatable infections (human papillomavirus [HPV] infection, human immunodeficiency virus [HIV] infection, herpes simplex virus [HSV] infection, and hepatitis B) and four curable infections (trichomonasis, chlamydiasis, gonorrhea, and syphilis).^3^ Global epidemiological data for STDs states that the incidence of STDs is very high in several countries, especially among individuals between the age of 15 to 50 years.^3^ Additionally, more than one million new cases of potentially curable STIs, mostly asymptomatic, are acquired on daily basis. It is further reported that 376 million new infections occur every year, involving at least one of the four curable STIs (gonorrhea, syphilis, chlamydiasis, and trichomoniasis). Of these, the burden of trichomonasis is the highest, with over 150 million new cases annually, closely followed by chlamydia, gonorrhea, and syphilis at 127 million, 87 million, and 6.3 million, respectively.^3^ Human papillomavirus (HPV) is the world's most prevalent sexually transmitted viral disease.^4^ In the United States of America alone, the burden of HPV infection is 80–110 million total cases with 14–20 million cases reported each year.^5^, ^6^

Oral sex is a form of sexual activity which involves the use of the mouth, teeth, lips, tongue or throat to stimulate the genitalia.^7^ It is a common practice among sexually active adults of all ages, socioeconomic status, sexual orientations, and races.^8^, ^9^, ^10^, ^11^, ^12^ This route of sexual contact can pose similar risks as other sexual routes such as vagina and anal sex.^1^ People may engage in oral sex (anlingus, fellatio, or cunninlingus) as part of foreplay before sexual intercourse, or during/after penetrative vaginal/anal intercourse.^7^ Therefore, oral sex is a potent route of transmission of syphilis,^13^ gonorrhea,^14^, ^15^ HSV,^16^ HIV,^17^ chlamydia,^18^ and HPV^15^ infections. Sexually transmitted oral infections, caused by carcinogenic pathogens, particularly HPV, is an emerging public health problem negatively impacting the field of oral health due to its ability to cause oropharyngeal cancer.^19^ Consequently, dental care professionals (DCPs), including dental surgeons, dental therapists, and dental hygienists, must play an important role in the prevention of oral STDs (i.e., oral infections contracted through oral sex) in dental patients.

With the persistently high burden of STDs, particularly those transmitted orally, in Africa, it is very crucial to evaluate the existing preventative interventions used by DCPs in preventing the problem, as this information will provide deep insights on the current landscape concerning this public health issue in Africa.^3^ However, the volume of studies, as well as the depth and breadth of the available evidence, on preventative interventions by DCPs in Africa on oral HPV, gonorrheal, chlamydial, syphilitic, and trichomonas infections is currently unknown; hence, the justification for this scoping review. This study therefore aimed at reviewing the existing literature on the preventative interventions of DCPs in Africa on oral HPV, gonorrheal, chlamydial, syphilitic, and trichomonas infections to identify the landscape of this area of research interest.

## 2 METHODS

### 2.1 Design

This scoping review adopted the research design proposed by Arksey and O'Malley^20^ and the AMSTAR 2 guideline informed the methodological and reporting process, to ensure quality.^21^ Also, the Preferred Reporting Items for Systematic Reviews and Meta‐analysis extension for conducting Scoping Reviews (PRISMA‐ScR) checklist guided the presentation of this review.^22^

### 2.2 Review question

Like other scoping reviews,^23^, ^24^ this current review seeks to answer this research question: *what is the available evidence in Africa preventative interventions, by DCPs, on oral HPV, gonorrheal, chlamydial, syphilitic and trichomonas infections?*

### 2.3 Identification of literatures

In addressing the scoping review question, a systematic search of 10 major international electronic research databases—SCOPUS, PubMed, Allied and Complementary Medicine Database (AMED), CINAHL Complete, CINAHL Ultimate, APA PsycArticles, APA PsycInfo, Dentistry and Oral Sciences Source, SPORTDiscus with Full Text, and Psychology and Behavioral Sciences Collection—was conducted on February 13, 2023 to scoop out literatures relevant to the review question. The search was conducted with the use of a combination of relevant search terms (aided by Boolean operators and truncations) which were developed through the PCC (P–Population; C–Concept; C–Context) framework.^25^ Based on this framework,^25^ the population of interest was dental patients; the concept was health interventions, by DCPs (dentists, dental specialists, dental hygienists, dental therapists, etc), on oral HPV, gonorrheal, chlamydial, syphilitic and trichomonas infections; and the context was Africa which comprised of 54 countries, 2 dependencies and 2 territories.^23^ Appendix: Tables A1, A2, A3 show the search strings obtained from the scoping search of these 10 databases.

### 2.4 Selection of literature

The literature retrieved from the database search was downloaded from these databases and imported to Rayyan software for deduplication.^26^ All duplicate records were expunged, and the residual literature was screened for inclusion in the scoping review based on a set of criteria highlighted below:

Inclusion criteria

- Literature published in peer‐reviewed journals.
- Literature published in English.
- Literature reporting empirical findings.
- Literature reporting preventative interventions by DCPs on oral HPV, gonorrheal, chlamydial, syphilitic, and trichomonas infections.
- Literature adopting any research design.
- Literature on studies conducted among dental patients in Africa.
- Literature with accessible full text.

Exclusion criteria

- Literature that was not published in peer‐reviewed journals (e.g., books, book chapters, etc).
- Literature not published in English.
- Literature that did not report empirical findings.
- Literature reporting preventative interventions by non‐DCPs on oral HPV, gonorrheal, chlamydial, syphilitic, and trichomonas infections.
- Literature on studies conducted among dental patients outside Africa.
- Literature on studies conducted among nondental patients in Africa.
- Literature with inaccessible full text. A literature is considered to have inaccessible full text if the authors could not retrieve such full text from the British Inter‐Library Loan or the corresponding author of such literature.

The screening process was in two stages. The first stage involved title and abstract screening to exclude obviously nonrelevant literature. Only the literature excluded in the first stage was screened at the second stage. In the second stage, screening of full text was done. Only the literature that met the above‐highlighted inclusion criteria were considered eligible for inclusion into the scoping review.

### 2.5 Data charting, collation, and summarization

In scoping reviews, data is extracted, collated, and summarized from the included literatures, provided they available.^23^, ^24^ However, this was not accomplished in this scoping review because no literature was found eligible for inclusion into the review. Hence, this is an empty scoping review.

## 3 RESULTS

**Figure 1:**
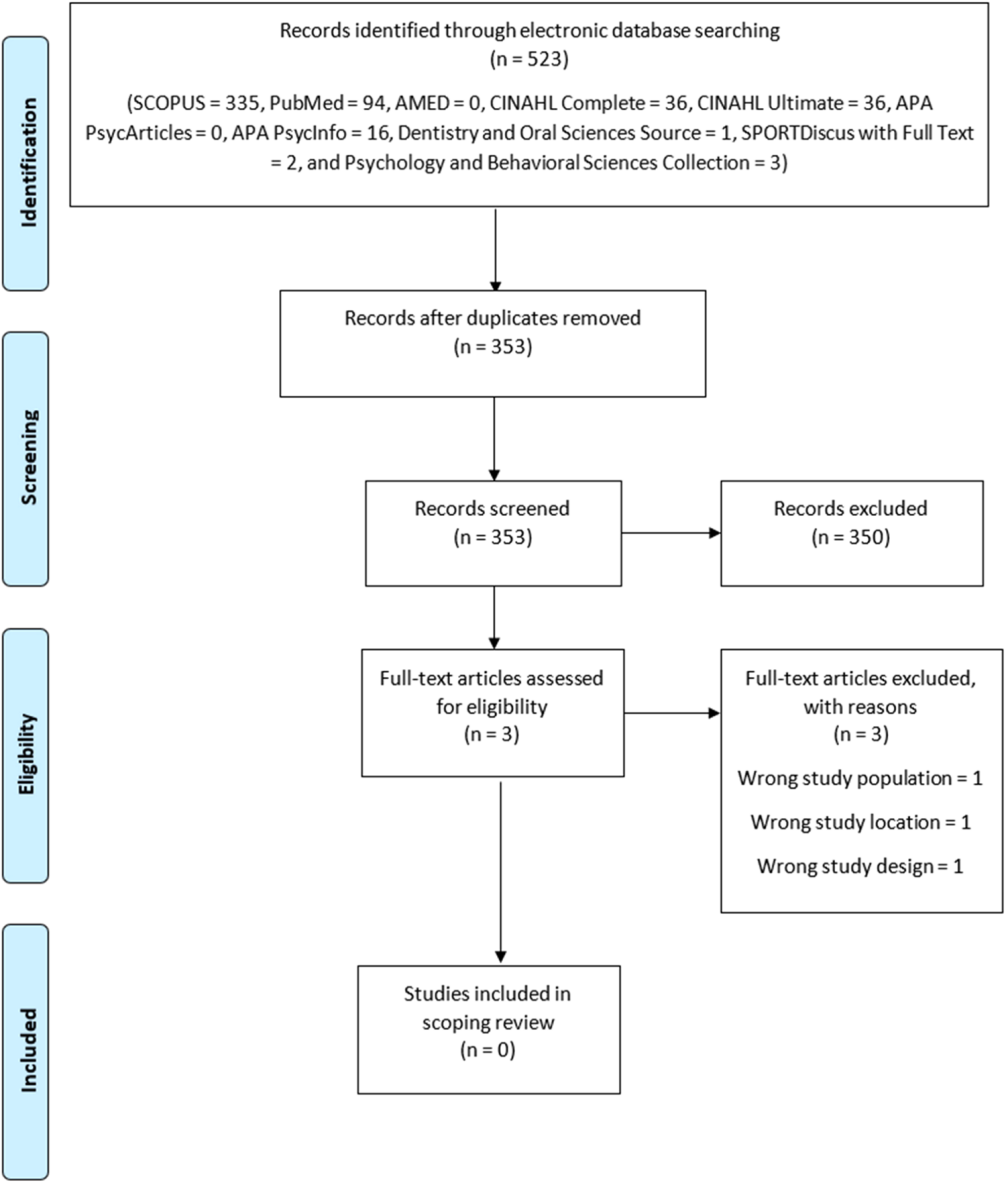
Flow chart diagram.

Five hundred and twenty‐three articles (SCOPUS = 335, PubMed = 94, AMED = 0, CINAHL Complete = 36, CINAHL Ultimate = 36, APA PsycArticles = 0, APA PsycInfo = 16, Dentistry and Oral Sciences Source = 1, SPORTDiscus with Full Text = 2, and Psychology and Behavioral Sciences Collection = 3) were retrieved from the electronic database search. After deduplication, 170 articles were excluded. The remaining 353 literature were subjected to screening, of which no relevant literature was found eligible for inclusion into the scoping review (Figure 1).

Table 1 shows the list of the literature whose full texts were screened in this review. The paper by Martini et al.^27^ was a case report on a DCP‐led intervention in Italy (Europe) on a Nigerian sex worker with oral HPV infection. The paper by Muzyka et al.^28^ was a prospective cohort study on pregnant females living with HIV in rural Malawi; however, the study did not involve a DCP‐led intervention. The paper by Masiiwa and Naidoo^29^ was a before and after study on DCP‐led intervention on HIV‐infected patients; however, the study population characteristics did not indicate that the studied population were patients who contracted oral diseases/infections through oral sex. Based on the review's selection criteria, none of these papers was included into this scoping review.

**Table 1:** List of articles with full text screened and their screening outcome.

			Outcome	
No.	Citation	Reference	Include	Exclude (reason)
1	Martini S, Colella G, Masiello A, Lanza A, Pisapia R, Cascone A, Di Martino F, Filippini A, Filippini P. HPV oral infection. Case report of an HIV‐positive Nigerian sex worker. Infez Med. 2007 Jun;15(2):115‐8. PMID: 17598999.	[27]	No	Yes (wrong study location)
2	Muzyka BC, Kamwendo L, Mbweza E, Lopez NB, Glick M, Matheson PB, Kershbaumer R, Nyrienda T, Malamud D, Constantine NT, Thompson J, Nyasulu Y, Saville R, Berthold P. Prevalence of HIV‐1 and oral lesions in pregnant women in rural Malawi. Oral Surg Oral Med Oral Pathol Oral Radiol Endod. 2001 Jul;92(1):56‐61. doi: 10.1067/moe.2001.112542. PMID: 11458246.	[28]	No	Yes (wrong study design)
3	Masiiwa A, Naidoo S. Oral lesions in HIV‐infected patients, before and after antiretroviral treatment. Southern African J Epidemiol Infect. 2011;26(4):271‐3.	[29]	No	Yes (wrong study population)

## 4 DISCUSSION

This is an empty scoping review of the preventative interventions by DCPs on oral STDs (with focus on HPV, gonorrheal, chlamydial, syphilitic, and trichomonas infections) in Africa. This empty review points out several concerns about the commonest oral STDs in Africa. Oral sex is commonly practised among heterosexuals and homosexuals in Africa,^30^, ^31^ but published interventions on the prevention and care of oral STDs, particularly in the dental care settings, are not available. This shows that oral STDs in Africa is a neglected public health area in Africa.

Pertinently, oral sex is commonly assumed to be a safer sexual practice when compared to genital‐genital sexual practices.^7^ However, as it is with other sexual practices, the exchange of body fluids often increases the risk of contracting oral STDs, especially when there is any form of cut or wound in the mouth, or genitalia.^7^, ^32^ It is also notable that the incidence of oral STDs is on the increase globally while the use of preventive measures against the disease is relatively low.^33^

In a recent survey of a sample of dental surgeons in Nigeria, Africa, it was observed that majority of them were unaware of the protective measures that can be used to prevent the transmission of oral STDs during oral sex^30^; hence, this may be a major factor contributing to the lack of preventative intervention, by DCPs, on oral STDs in Africa. Also, in the survey,^30^ it was identified that the practice of educating dental patients on the risks of unprotected oral sex as well as the precautionary measures that can be adopted to ensure safer oral sex was very low among participating dental surgeons.^30^ However, in the study,^30^ the low practice was attributed to the highly conservative sociocultural landscape on sex‐related issues in Nigeria. Based on these findings, it can be suggested that the lack of empirical research evidence on the preventative interventions by DCPs concerning oral STDs is attributable to the perception of oral sex as a taboo in African culture, and inadequate knowledge of DCPs in Africa on protective measures concerning oral sex.^30^

It has been documented in the literature that STDs can manifest in the mouth, even if they were not transmitted through the oral route.^34^ Also, STDs contracted through nonoral sex route (vaginal, penile, or anal route) can be transmitted to another person through oral sex, causing oral STD.^34^ For example, syphilitic oral lesions are highly contagious; hence, such lesions could enhance oral transmission of STDs during unprotected oral sex.^34^ Unfortunately, the prevalence rates of unprotected oral sex in several African countries are high^31^; however, this finding is similar to that observed elsewhere.^35^ For example, in a study, by Strome et al.^35^ it was reported that less than 10% of a sampled youth population in the USA ever practised protected oral sex. Overall, this shows that unprotected oral sex is a common practice in several countries of the world; hence, the practice is a global sexual health problem. However, Africa has one of the greatest share of the STD disease burden.^3^, ^30^, ^31^

## 5 CLINICAL RELEVANCE

The findings obtained from this empty scoping review indicates that there has not been adequate research engagement of DCPs in Africa in the prevention of oral STDs. However, it is also possible that such engagements might exist in Africa, but they have not been captured with adequate research. Hence, this is a research gap that needs to be filled, as evidence on this public health area is very crucial for the development, implementation, and evaluation of public health policies on sexual health promotion and oral STD prevention in Africa.

## 6 CONCLUSION

This is an empty scoping review of the preventative interventions by DCPs on oral HPV, gonorrheal, chlamydial, syphilitic, and trichomonas infections in Africa. This empty scoping review points to several concerns including low research about oral STDs in Africa. This empty scoping review also suggests that the limited knowledge of oral STDs among DCPs in Africa might also account for the low involvement of DCPs in Africa on intervention‐based research on oral STD prevention. Finally, this review recommends the need for more intervention‐based research by DCPs in Africa on this neglected topic area.

## AUTHOR CONTRIBUTIONS

**Kehinde K. Kanmodi**: Conceptualization; data curation; formal analysis; funding acquisition; investigation; methodology; project administration; resources; software; supervision; validation; visualization; writing—original draft; writing—review & editing. **Jimoh Amzat**: Resources; writing—original draft; writing—review & editing. **Afeez A. Salami**: Data curation; investigation; methodology; resources; software; validation. **Eyinade A. Egbedina**: Data curation; investigation; resources; software. **Ramat O. Braimah**: Resources; writing—original draft.

## CONFLICTS OF INTEREST STATEMENT

Kehinde Kazeem Kanmodi is an Editorial Board member of Health Science Reports and a co‐author of this article. To minimize bias, they were excluded from all editorial decision‐making related to the acceptance of this article for publication. The remaining authors declare no conflict of interest.

## TRANSPARENCY STATEMENT

The lead author Kehinde Kazeem Kanmodi affirms that this manuscript is an honest, accurate, and transparent account of the study being reported; that no important aspects of the study have been omitted; and that any discrepancies from the study as planned (and, if relevant, registered) have been explained.

## Data Availability

Data sharing is not applicable to this article as no new data were created or analyzed in this study.

## 1

**Table A1:** Search string for PubMed database search.

Tag	Subject search	Search string
#1	HPV, gonorrheal, chlamydial, trichomonas and syphilitic infections	((((((((((Sexually transmitted infection[Title/Abstract]) OR (STI[Title/Abstract])) OR (sexually transmitted disease[Title/Abstract])) OR (STD[Title/Abstract])) OR (human papillomavirus[Title/Abstract])) OR (HPV[Title/Abstract])) OR (gonorrhea[Title/Abstract])) OR (gonorrhea[Title/Abstract])) OR (chlamydia*[Title/Abstract])) OR (syphili*[Title/Abstract])) OR (trichomon*[Title/Abstract])
#2	Dental care professionals/dental patients	(((((((Dental) OR (oral)) OR (dentist*)) OR (maxillofacial)) OR (orthodonti*)) OR (prosthodonti*)) OR (prosthetic dentist*)) OR (periodont*)
#3	Interventions	((((((Intervention[Title/Abstract]) OR (trial[Title/Abstract])) OR (cohort[Title/Abstract])) OR (case control[Title/Abstract])) OR (before[Title/Abstract] AND after[Title/Abstract])) OR (experiment*[Title/Abstract])) OR (quasi experiment*[Title/Abstract])
#4	African countries, dependencies, and territories	(((((((((((((((((((((((((((((((((((((((((((((((((((((((((((Algeria[MeSH Terms]) OR (Angola[MeSH Terms])) OR (Benin[MeSH Terms])) OR (Botswana[MeSH Terms])) OR (burkina faso[MeSH Terms])) OR (burundi[MeSH Terms])) OR (cabo verde[MeSH Terms])) OR (cape verde[MeSH Terms])) OR (cameroon[MeSH Terms])) OR (central african republic[MeSH Terms])) OR (chad[MeSH Terms])) OR (comoros[MeSH Terms])) OR (congo[MeSH Terms])) OR (ivory coast[MeSH Terms])) OR (cote d ivoire[MeSH Terms])) OR (djibouti[MeSH Terms])) OR (democratic republic of congo[MeSH Terms])) OR (egypt[MeSH Terms])) OR (equatorial guinea[MeSH Terms])) OR (eritrea[MeSH Terms])) OR (eswatini[MeSH Terms])) OR (ethiopia[MeSH Terms])) OR (gabon[MeSH Terms])) OR (gambia[MeSH Terms])) OR (ghana[MeSH Terms])) OR (guinea[MeSH Terms])) OR (guinea bissau[MeSH Terms])) OR (kenya[MeSH Terms])) OR (lesotho[MeSH Terms])) OR (liberia[MeSH Terms])) OR (libya[MeSH Terms])) OR (madagascar[MeSH Terms])) OR (malawi[MeSH Terms])) OR (mali[MeSH Terms])) OR (mauritania[MeSH Terms])) OR (mauritius[MeSH Terms])) OR (morocco[MeSH Terms])) OR (mozambique[MeSH Terms])) OR (namibia[MeSH Terms])) OR (niger[MeSH Terms])) OR (nigeria[MeSH Terms])) OR (rwanda[MeSH Terms])) OR (sao tome and principe[MeSH Terms])) OR (senegal[MeSH Terms])) OR (seychelles[MeSH Terms])) OR (sierra leone[MeSH Terms])) OR (somalia[MeSH Terms])) OR (south africa[MeSH Terms])) OR (south sudan[MeSH Terms])) OR (sudan[MeSH Terms])) OR (tanzania[MeSH Terms])) OR (togo[MeSH Terms])) OR (tunisia[MeSH Terms])) OR (uganda[MeSH Terms])) OR (zambia[MeSH Terms])) OR (zimbabwe[MeSH Terms])) OR (reunion[MeSH Terms])) OR (saint helena[MeSH Terms])) OR (western sahara[MeSH Terms])) OR (mayotte[MeSH Terms])
#5	#1 AND #2 AND #3 AND #4	(((#1) AND (#2)) AND (#3)) AND (#4)

**Table A2:** Search string for SCOPUS database search.

Tag	Subject search	Search string
#1	HPV, gonorrheal, chlamydial, trichomonas and syphilitic infections	(TITLE‐ABS‐KEY (sexually AND transmitted AND infection) OR TITLE‐ABS‐KEY (sti) OR TITLE‐ABS‐KEY (sexually AND transmitted AND disease) OR TITLE‐ABS‐KEY (std) OR TITLE‐ABS‐KEY (human AND papillomavirus) OR TITLE‐ABS‐KEY (gonorrhea) OR TITLE‐ABS‐KEY (gonorrhea) OR TITLE‐ABS‐KEY (chlamydia*) OR TITLE‐ABS‐KEY (syphili*) OR TITLE‐ABS‐KEY (trichomon*))
#2	Dental care professionals/dental patients	(TITLE‐ABS‐KEY (dental) OR TITLE‐ABS‐KEY (oral) OR TITLE‐ABS‐KEY (dentist*) OR TITLE‐ABS‐KEY (maxillofacial) OR TITLE‐ABS‐KEY (orthodonti*) OR TITLE‐ABS‐KEY (prosthodonti*) OR TITLE‐ABS‐KEY (prosthetic AND dentist*) OR TITLE‐ABS‐KEY (periodont*))
#3	Interventions	(TITLE‐ABS‐KEY (intervention) OR TITLE‐ABS‐KEY (trial) OR TITLE‐ABS‐KEY (cohort) OR TITLE‐ABS‐KEY (case AND control) OR TITLE‐ABS‐KEY (before AND after) OR TITLE‐ABS‐KEY (experiment*) OR TITLE‐ABS‐KEY (quasi AND experiment*))
#4	African countries, dependencies, and territories	((TITLE‐ABS‐KEY (angola) OR TITLE‐ABS‐KEY (benin) OR TITLE‐ABS‐KEY (botswana) OR TITLE‐ABS‐KEY (burkina AND faso) OR TITLE‐ABS‐KEY (burundi) OR TITLE‐ABS‐KEY (cameroon) OR TITLE‐ABS‐KEY (cabo AND verde) OR TITLE‐ABS‐KEY (cape AND verde) OR TITLE‐ABS‐KEY (central AND african AND republic) OR TITLE‐ABS‐KEY (chad) OR TITLE‐ABS‐KEY (comoros) OR TITLE‐ABS‐KEY (congo) OR TITLE‐ABS‐KEY (ivory AND coast) OR TITLE‐ABS‐KEY (democratic AND republic AND of AND congo) OR TITLE‐ABS‐KEY (djibouti) OR TITLE‐ABS‐KEY (equatorial AND guinea) OR TITLE‐ABS‐KEY (eritrea) OR TITLE‐ABS‐KEY (ethiopia) OR TITLE‐ABS‐KEY (gabon) OR TITLE‐ABS‐KEY (gambia) OR TITLE‐ABS‐KEY (ghana) OR TITLE‐ABS‐KEY (guinea) OR TITLE‐ABS‐KEY (guinea‐bissau) OR TITLE‐ABS‐KEY (kenya) OR TITLE‐ABS‐KEY (lesotho) OR TITLE‐ABS‐KEY (liberia) OR TITLE‐ABS‐KEY (madagascar) OR TITLE‐ABS‐KEY (malawi) OR TITLE‐ABS‐KEY (mali) OR TITLE‐ABS‐KEY (mauritania) OR TITLE‐ABS‐KEY (mauritius) OR TITLE‐ABS‐KEY (mayotte) OR TITLE‐ABS‐KEY (mozambique) OR TITLE‐ABS‐KEY (namibia) OR TITLE‐ABS‐KEY (niger) OR TITLE‐ABS‐KEY (nigeria) OR TITLE‐ABS‐KEY (reunion) OR TITLE‐ABS‐KEY (rwanda) OR TITLE‐ABS‐KEY (saint AND helena) OR TITLE‐ABS‐KEY (sao AND tome AND principe) OR TITLE‐ABS‐KEY (senegal) OR TITLE‐ABS‐KEY (seychelles) OR TITLE‐ABS‐KEY (sierra AND leone) OR TITLE‐ABS‐KEY (somalia) OR TITLE‐ABS‐KEY (south AND africa) OR TITLE‐ABS‐KEY (south AND sudan))) OR ((TITLE‐ABS‐KEY (eswatini) OR TITLE‐ABS‐KEY (togo) OR TITLE‐ABS‐KEY (uganda) OR TITLE‐ABS‐KEY (zambia) OR TITLE‐ABS‐KEY (zimbabwe) OR TITLE‐ABS‐KEY (egypt) OR TITLE‐ABS‐KEY (libya) OR TITLE‐ABS‐KEY (algeria) OR TITLE‐ABS‐KEY (tunisia) OR TITLE‐ABS‐KEY (morocco) OR TITLE‐ABS‐KEY (western AND sahara) OR TITLE‐ABS‐KEY (sudan) OR TITLE‐ABS‐KEY (tunisia)))
#5	#1 AND #2 AND #3 AND #4	(#1) AND (#2) AND (#3) AND (#4)

**Table A3:** Search string for other database (AMED–The Allied and Complementary Medicine Database; CINAHL Complete; Dentistry and Oral Sciences Source; SPORTDiscus with Full Text; APA PsycArticles; Psychology and Behavioral Sciences Collection; APA PsycInfo and CINAHL Ultimate) search via EBSCO interface.

Tag	Subject search	Search string
S1	HPV, gonorrheal, chlamydial, trichomonas and syphilitic infections	AB Sexually transmitted infection OR AB STI OR AB sexually transmitted disease OR AB STD OR AB human papillomavirus OR AB HPV OR AB gonorrhea OR AB gonorrhea OR AB chlamydia* OR AB syphili* OR AB trichomon*
S2	Dental care professionals/dental patients	AB dental OR AB oral OR AB dentist* OR AB maxillofacial OR AB orthodonti* OR AB prosthodonti* OR AB prosthetic dentist* OR AB periodont*
S3	Interventions	AB intervention OR AB trial OR AB cohort OR AB case control OR AB (before and after) OR AB experiment* OR AB quasi experiment*
S4	African countries, dependencies, and territories	AB algeria OR AB angola OR AB benin OR AB botswana OR AB burkina faso OR AB burundi OR AB cape verde OR AB cabo verde OR AB cameroon OR AB central african republic OR AB chad OR AB comoros OR AB congo OR AB cote d'ivoire OR AB ivory coast OR AB djibouti OR AB democratic republic of congo OR AB egypt OR AB equatorial guinea OR AB eritrea OR AB eswatini OR AB ethiopia OR AB gabon OR AB gambia OR AB ghana OR AB guinea OR AB guinea bissau OR AB kenya OR AB lesotho OR AB liberia OR AB libya OR AB madagascar OR AB malawi OR AB mali OR AB mauritania OR AB mauritius OR AB morocco OR AB mozambique OR AB namibia OR AB niger OR AB nigeria OR AB rwanda OR AB (sao tome and principe) OR AB senegal OR AB seychelles OR AB sierra leone OR AB somalia OR AB south Africa OR AB south sudan OR AB sudan OR AB tanzania OR AB togo OR AB tunisia OR AB uganda OR AB zambia OR AB zimbabwe OR AB reunion OR AB saint helena OR AB western sahara OR AB mayotte
S5	S1 AND S2 AND S3 AND S4	S1 AND S2 AND S3 AND S4
